# Versatility of the O-Z flap for back reconstruction after giant basal cell carcinoma resection: A case report and review of the literature

**DOI:** 10.1016/j.ijscr.2019.08.034

**Published:** 2019-09-11

**Authors:** Matteo Torresetti, Matteo Gioacchini, Alessandro Scalise, Giovanni Di Benedetto

**Affiliations:** Clinic of Plastic and Reconstructive Surgery, Department of Experimental and Clinical Medicine, Marche Polytechnic University Medical School, Via Conca 71, 60126, Ancona, Italy

**Keywords:** Giant basal cell carcinoma, Back reconstruction, Flap, O-Z flap

## Abstract

**Introduction:**

Giant basal cell carcinomas are rare and potentially aggressive skin malignancies that are infrequently reported in the Literature, and they usually require aggressive surgical resection and immediate soft tissue reconstruction with skin grafts or flaps. The large size of GBCCs has relevant implications not only for the metastatic potential, but even for the reconstructive challenging due to the possible limited availability of tissues for flap coverage.

**Presentation of case:**

We report the peculiar case of an old patient who underwent to GBCC resection of the back measuring 16 × 13 cm, and immediate reconstruction with a large O to Z flap harvested from the back; one-year follow-up examination revealed a satisfactory result and no recurrence of the disease was observed.

**Discussion:**

The review of the Literature showed that reconstructive options are mainly determined by the site and extent of the defect, exposed structures and patient characteristics and comorbidities, and previous series reported the use of skin grafts only, pedicled myocutaneous or perforator flaps, exclusively free flaps and a combination of techniques. Therefore, in this scenario local flaps such as O-Z flaps are less invasive procedures, that usually have lower rate of postoperative complications and shorter hospitalization compared to more complex procedures.

**Conclusions:**

Despite the use of O-Z flap is quite unusual for back reconstruction, we believe that this flap represents a valid and safe alternative in selected cases, particularly in the elderly and medically compromised patients with multiple comorbidities.

## Highlights

• Giant basal cell carcinomas are rare and potentially aggressive tumors.
• Aggressive surgical resection with suggested wide free margins is suggested.
• Soft tissue reconstruction for back lesions may be challenging.
• The O-Z flap represents a safe and reliable alternative for back reconstruction.

## 1 Background

Giant basal cell carcinomas (GBCCs) represent a quite rare oncological entity. According to the American Joint Committee on Cancer, GBCC is defined as a tumor with a diameter larger than 5 cm.

Contrary to their more common counterpart (BCCs), GBCCs have a more aggressive biological behavior, with deep tissue invasion and involvement of extradermal structures such as muscles and bones, as well as by metastasis and commonly have a poor prognosis. Tumors larger than 10 cm in diameter have an incidence of metastasis and/or fatal outcome of 45%. They are infrequently reported in the Literature with an occurrence rate of 0.5%–1% out of all types of BCCs, and only few case report or case series have been described [1].

The most frequent anatomical site is the trunk, especially on the back, where they go unnoticed by the patient and can be hidden by clothes. Despite no established guidelines for GBCCs treatment exist, given its rarity, the mainstay of treatment is aggressive surgical resection with suggested adequate margin range of 2.5–3 cm, and immediate soft tissue reconstruction with grafting or flaps [[1], [2], [3]].

We report the case of a patient who underwent to GBCC resection of the back and immediate reconstruction with a large O-Z flap; one-year follow-up examination revealed a satisfactory result and no recurrence of the disease was observed.

In our opinion this flap represents a safe and reliable alternative to other more complex techniques for back reconstruction, and to our knowledge it’s the first report of this flap for this kind of reconstruction. The present work has been reported in line with the SCARE criteria [4].

## 2 Case presentation

**Fig. 1:**
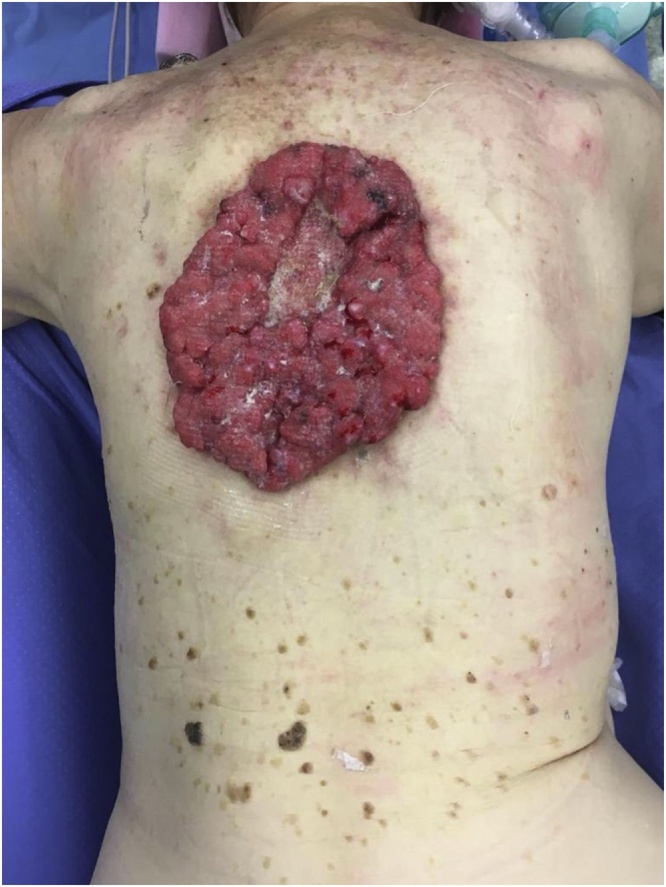
Preoperative view of the giant basal cell carcinoma of the back measuring 16 × 13 cm.

A 88-year-old woman was admitted to our hospital for a large fungating lesion on her upper back measuring 16 × 13 cm (Fig. 1). The lesion had been present for the last 8 years but the patient had no sought for medical attention, and it was incidentally found when the patient was taken to her general practitioner for increasing fatigue. Physical examination revealed a bleeding lesion with granulation tissue and copious serous drainage. Blood test findings revealed severe iron deficiency anemia (Hb 6.8 g/dl) and hypoproitenemia (Albumin 1.8 g/dl), probably due to intermittent bleeding of the tumor and serum loss. Preoperative punch biopsies of the lesion were positive for Basal cell carcinoma, and total body computed tomography (CT) scanning demonstrated the mass to be confined to the skin and subcutaneous fat while no evidence of lymphadenopathy or metastatic disease was observed.

**Fig. 2:**
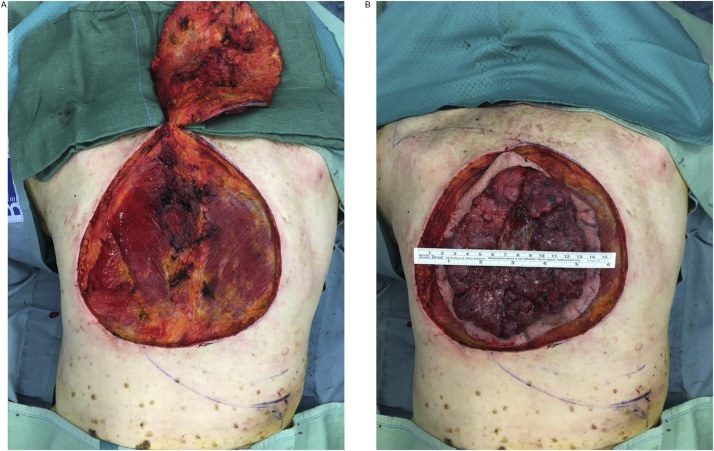
Intraoperative figure showing the defect after tumor excision (A) and the sample sent to pathologist with flap markings (B).

Therefore the patient underwent wide local excision of the BCC with a 2 cm margin up to the skeletal muscle after a multidisciplinary tumor board consultation, and an immediate reconstruction by using an O-Z flap, harvested from the back, was finally achieved (Fig. 2). Histopathology revealed no residual BCC in the tumor bed and the patient was discharged 4 days postoperative.

**Fig. 3:**
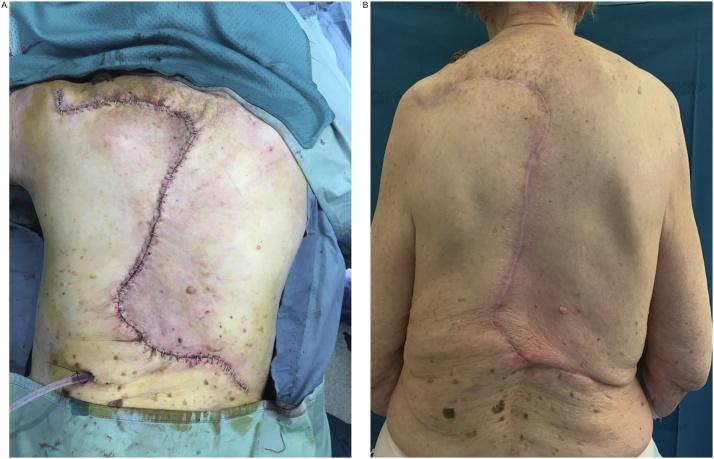
Immediate postoperative result after the O-Z flap reconstruction (A) and the one-year follow-up picture showing no recurrences of the disease and an acceptable aesthetic result (B).

One-year follow-up examination showed no recurrences of the disease and an acceptable aesthetic and functional result (Fig. 3).

## 3 Discussion

GBCCs are rare and potentially aggressive skin malignancies typical of neglected patients, and they are frequently diagnosed secondary to other medical problems such as anemia, hypoproteinemia, infections or sepsis [1,2,5].

Aggressive surgical resection with the main goal of obtaining free margins, preventing metastasis, and an immediate 1-stage reconstruction of the involved structures such as bone and soft tissues are mandatory [[1], [2], [3],^6^]. The large size of GBCCs has relevant implications not only for the metastatic potential, but even for the reconstructive challenging. Reconstructive options are mainly determined by the site and extent of the defect, exposed structures and patient characteristics and comorbidities [7,8], and previous series reported the use of skin grafts only, pedicled myocutaneous or perforator flaps, exclusively free flaps and a combination of techniques [7].

Lackey et al. in 2007 reported a series of 8 challenging cases, but only one patient reported a chest wall reconstruction with a TRAM flap [7].

Archontaki et al. in 2009 reviewed 48 cases of GBCC throughout the Literature and they found 13 cases of back lesions [1], but only one study described a flap reconstruction by using a myocutaneous flap [5]. In their personal series, Archontaki et al. described one back complex reconstruction with a bilateral myocutaneous latissimus dorsi flap and a fasciocutaneous gluteal flap.

Bogdanić et al. in 2009 studied a single case of GBCC of the back treated by excision and skin grafts [9].

Sinha et al. in 2013 described a single case of GBCC of the back which was reconstructed with a double perforator flap [8].

Wagner et al. in 2016 reported a GBCC of the upper back managed with radiation therapy alone due to the wide extension of the lesion [10].

Therefore surgical repair of such soft tissue defects of the back is often challenging and usually requires complex techniques due to the inadequate availability of local soft tissue coverage. Sometimes microsurgical reconstruction by using free flaps or pedicled perforator flaps may be difficult, particularly in those elderly and medically compromised patients with multiple comorbidities. Myocutanoues flaps such as latissimus dorsi or TRAM flap provide excellent soft tissue reconstruction, even if the possible functional sequelae should be considered. Skin grafts may provide an adequate coverage even for large defects; nevertheless they are unsuitable for deeper defects with bone exposure.

Therefore, in this scenario local flaps such as O-Z flaps are less invasive procedures, that usually cause minor blood loss, shorter surgical duration, lower rate of postoperative complications and shorter hospitalization compared to microsurgical flaps. They are simple and reliable techniques with a short learning curve, and they do not require microsurgical skills. Moreover, the O-Z flap provides a vascularized coverage even for large and deep defects, thus allowing at the same time a wide tumor excision and a lower risk of recurrences. Another advantage is the low donor site morbidity, as no skin grafting or multiple flaps are necessary for donor site wound closure. Finally, if compared to myocutaneous flaps, the O-Z flap is a muscle-sparing technique, with important consequences from a functional perspective.

The O-Z flap is a double-rotation flap typically used in the repair of circular to oval defects on the scalp, forehead, and temple. As its design provides minimal distortion of nearby structures and tissue conservation, it is mechanically simple with predictable tension vectors [11].

We encourage its use particularly for those lesions located in the midline area of the back, with consequent available tissue on opposite sides of the defect, thus allowing an optimal flap design. Furthermore, elderly patients with skin excess and laxity are more suitable for this kind of reconstruction in the midline area, that could usually be exposed to a higher tension stress.

## 4 Conclusions

GBCCs usually require aggressive surgical management and soft tissues reconstruction may be challenging. We propose the use of the O-Z flap as a safe and simple alternative for back reconstruction. In our opinion this flap could be useful in those selected cases where a more complex procedure is not recommended.

## Funding

The authors received no financial support for the research, authorship, and/or publication of this article.

## Ethical approval

The present study is exempt from ethical approval in our institution.

## Consent

Written informed consent was obtained from the patient for publication of this case report and accompanying images. A copy of the written consent is available for review by the Editor-in-Chief of this journal on request

## Author’s contribution

Dr. Matteo Torresetti participated to surgical procedure, led the study design, data collection and interpretation and wrote the paper.

Dr. Matteo Gioacchini performed the surgical procedure and participated to study design.

Dr. Alessandro Scalise participated to study design and data collection and interpretation.

Prof. Giovanni Di Benedetto participated to study design, approved and drafted the final manuscript.

## Registration of research studies

None.

## Guarantor

Dr. Matteo Torresetti.

Dr. Matteo Gioacchini.

Dr. Alessandro Scalise.

Prof. Giovanni Di Benedetto.

## Provenance and peer review

Not commissioned, externally peer-reviewed.

## Declaration of Competing Interest

The authors declare that there is no conflict of interest.

## References

[1] Archontaki M., Stavrianos S.D., Korkolis D.P., Arnogiannaki N., Vassiliadis V., Liapakis I.E., Christ H., Rapidis A.D., Kokkalis G. (2009). Giant basal cell carcinoma: clinicopathological analysis of 51 cases and review of the literature. Anticancer Res..

[2] Santos P.J., Prendergast C., Leis A. (2016). Giant anterior chest wall basal cell carcinoma: an approach to palliative reconstruction. Case Rep. Oncol. Med..

[3] Warbrick-Smith J., O’Neill J.K., Wilson P. (2013). Giant anterior chest wall basal cell carcinoma: a reconstructive challenge and review of the literature. BMJ Case Rep..

[4] Agha R.A., Borrelli M.R., Farwana R., Koshy K., Fowler A., Orgill D.P., For the SCARE group (2018). The SCARE 2018 statement: updating consensus surgical case report (SCARE) guidelines. Int. J. Surg..

[5] de Bree E., Laliotis A., Manios A., Tsiftsis D.D., Melissas J. (2010). Super giant basal cell carcinoma of the abdominal wall: still possible in the 21st century. Int. J. Dermatol..

[6] Araco A., Gravante G., Araco F., Delogu D., Cervelli V. (2006). Giant basal cell carcinomas. Plast. Reconstr. Surg..

[7] Lackey P.L., Sargent L.A., Wong L., Brzezienski M., Kennedy J.W. (2007). Giant basal cell carcinoma surgical management and reconstructive challenges. Ann. Plast. Surg..

[8] Sinha S., Yip M.J., Gill S., Pohl M.J., Donahoe S.R. (2013). A giant fungating metastatic basal cell carcinoma of the back and novel reconstruction using two large keystone design island perforator flaps. J. Plast. Reconstr. Aesthet. Surg..

[9] Bogdanić B., Smud S., Bagatin D., Nola M., Mijatović D., Majerović M. (2009). Giant basal cell carcinoma of the back: a case report and review of the literature. Coll. Antropol..

[10] Wagner R.D., Nguyen H.P., Tyring S.K. (2016). Giant ulcerative lesion on the upper back: using a differential diagnosis to formulate a clinical approach. Einstein (Sao Paulo).

[11] Regula C.G., Liu A., Lawrence N. (2016). Versatility of the O-Z flap in the reconstruction of facial defects. Dermatol. Surg..

